# Kansas

**Published:** 1897-08

**Authors:** 


*Kansas* proposes to try, by statute, to fix the value of animals killed by railroads, and by legislative enactment, also, to punish those who commit rape by having all who are found guilty castrated.

